# Comparative Assessment of the Potential of *Xylocoris flavipes* (Hemiptera: Anthocoridae) and Two *Cheyletus* spp. (Trombidiformes: Cheyletidae) for Managing *Liposcelis decolor* (Psocodea: Liposcelididae)

**DOI:** 10.3390/insects17030332

**Published:** 2026-03-18

**Authors:** Augustine Bosomtwe, James Danso, George Opit, Brad Kard, Kristopher Giles, Carla Goad

**Affiliations:** 1 127 Noble Research Center, Department of Entomology and Plant Pathology, Oklahoma State University, Stillwater, OK 74078, USA; george.opit@okstate.edu (G.O.); b.kard@okstate.edu (B.K.); kris.giles@okstate.edu (K.G.); 2 CSIR—Plant Genetic Resources Research Institute, Bunso P.O. Box 7, Ghana; 3 Agricultural Research Station, Fort Valley State University, 1005 State University Drive, Fort Valley, GA 31030, USA; james.danso@fvsu.edu; 4 Department of Statistics, Oklahoma State University, 301, Mathematics, Statistics and Computer Sciences, Stillwater, OK 74078, USA; carla.goad@okstate.edu

**Keywords:** psocid, biological control, warehouse pirate bug, predatory mite, pest management

## Abstract

Psocids are difficult to manage using insecticides, hence the need for alternatives including biological control. Evaluation of data from two separate studies was conducted. One study investigated the potential of *Cheyletus eruditus* (Shrank) (Trombidiformes: Cheyletidae) and *Cheyletus malaccensis Oudemans* to manage *Liposcelis decolor* (Pearman) (Psocodea: Liposcelididae), whereas the other investigated the potential of *Xylocoris flavipes* (Reuter) (Hemiptera: Anthocoridae) to do the same. Temperature and relative humidity conditions were similar in both studies. However, the five predator–prey (P-P) ratios for the mites (0:20, 1:20, 2:20, 4:20 and 10:20) were different from those of *X. flavipes* (0:240, 1:240, 2:240, 3:240 and 5:240). The three predators demonstrated significant prey suppression; however, the level of control by *X. flavipes* was higher compared to the mites. At optimal prey conditions of 32 °C and 75% RH, all predators maintained high suppression. Temperature significantly influenced progeny production, with high reproduction observed at 20 and 24 °C for the *Cheyletus* spp. and at 28 and 32 °C for *X. flavipes*. Relative humidity of 63% was detrimental to *Cheyletus* spp. progeny production. While the results from this evaluation for the purpose of comparison should be interpreted cautiously, the different performances of the predators provide valuable insights for biological control of stored-product psocids.

## Simple Summary

Managing psocids with conventional insecticides is difficult due to tolerance and resistance. This study evaluated three predator species—two predatory mites, *Cheyletus eruditus* and *Cheyletus malaccensis*, and the warehouse pirate bug, *Xylocoris flavipes*—to determine their potential for managing *Liposcelis decolor*, a psocid species with high tolerance to phosphine. The comparison used data from two separate laboratory studies conducted under similar temperature and humidity conditions but with different predator-to-prey ratios. All three predators effectively reduced *L. decolor* populations, with *X. flavipes* achieving the highest prey suppression—above 97% across all environmental conditions tested. *Xylocoris flavipes* also tolerated a wider range of relative humidity levels, whereas *C*. *eruditus* and *C. malaccensis* required relative humidity above 63% to survive and reproduce. Temperature influenced predator progeny production differently. *Cheyletus eruditus* and *C. malaccensis* produced more offspring at cooler temperatures (20–24 °C), whereas *X. flavipes* reproduced best at warmer temperatures (28–32 °C) with lower predator–prey ratios. These studies provide baseline information on how environmental conditions influence predator performance, with *X. flavipes* demonstrating broader environmental tolerance suitable for diverse storage conditions. Suppression caused by the predators demonstrate their potential as a biological control agent to manage psocids.

## 1. Introduction

Stored-product psocids (Psocodea: Liposcelididae) are regarded as insect pests of economic importance worldwide [1,2]. They are capable of causing substantial weight losses of stored grains through direct consumption of endosperm and germ, contaminate commodities by distribution of molds, and transmit disease pathogens that are associated with human and animal health problems [3,4,5]. Psocid infestation also poses risk to international trade because of potential rejection of infested commodities [1,5,6]. Managing psocids using insecticides including phosphine, the main insecticide used to protect stored products against lepidopteran and coleopteran pests is difficult because many psocid species have natural tolerance and rapidly develop high levels of resistance [7,8]. Economically important psocid species including *Liposcelis decolor*, *L. bostrychophila* and *L*. *entomophila* have high tolerance to phosphine and recover quickly from poorly conducted phosphine fumigations [8,9,10]. Discriminating doses of phosphine for *L*. *decolor* in a recent study were 249.76 and 194.5 ppm over 20–h and 72–h of fumigations, respectively [8]. When compared with other common stored-product insect pests, these phosphine concentrations are high [8].

Natural enemies of stored grain pests including *Xylocoris flavipes* (Reuter) (Hemiptera: Anthocoridae), *Cheyletus malaccensis* Oudemans (Trombidiformes: Cheyletidae) and *Cheyletus eruditus* (Schrank) are some of the predators commonly associated with stored-product insect pests in post-harvest agricultural systems [7,11,12,13,14,15,16]. This natural enemy complex represents potential use for biological control against stored-product psocids [6,11,12,13]. The warehouse pirate bug, *X. flavipes* prey voraciously on the immature stages of stored-product moths and beetles [14,17,18]. Recent studies [13,19,20] showed *X*. *flavipes* has the potential to manage adults and nymphs of *L. decolor*, a psocid species with high tolerance to phosphine [8]. *Xylocoris flavipes* is a polyphagous predator that is widely distributed in processing and storage facilities and can naturally penetrate grain mass to locate their prey [11,16]. This predator can respond both functionally and numerically to increasing prey populations to achieve high prey suppression [13,14]. In the United States, *X*. *flavipes* is one of the Environmental Protection Agency (EPA)-approved bioagents for use against insect pests of stored-products [21]. *Cheyletus malaccensis* and *C. eruditus* are predatory mite species widely distributed in grain storage environments and feed on mite pests, the eggs and the larvae of insect pests of stored commodities [22,23,24]. Several studies showed that these two cheyletid mites are capable of suppressing *L. decolor* populations because they can survive on *L. decolor* to establish and increase their progeny production [12,25,26]. *Cheyletus malaccensis* is dominant in temperate storage conditions and has natural ability to penetrate bulk grains, has high fertility and tolerates wide temperature ranges [27,28]. However, *C*. *eruditus* is commonly associated with grain residues in both temperate and tropical conditions [29]. It is marketed as Cheyletin^®^ and is approved for managing mite pests in food storage systems [29].

The effectiveness of biocontrol agents in pest management can be influenced by interactions between abiotic and biotic factors within the storage environment [30,31]. Among abiotic factors, relative humidity and temperature are the critical variables that regulate the rate of reproduction, development and death, and influence arthropod population dynamics [26,32]. Predators of psocids, including *X*. *flavipes* and cheyletid mites, require varying temperature and relative humidity to survive and complete their development compared with their prey. Optimal conditions of *X. flavipes* range between 28 and 31 °C at 63–70% RH [33,34,35], whereas *C. malaccensis* can develop at temperatures between 11.6 and 37.8 °C [36,37,38] and the life cycle of *C. eruditus* can be completed at 12–35 °C and 60–90% RH [11]. Development of psocids from egg to adult can be completed at temperatures between 20 and 42.5 °C, with optimal conditions ranging between 32.5 and 35 °C and 70–80% RH [39,40,41]. In grain storage ecosystems, key biotic factors that influence predator–prey interactions include cannibalism, competition, interference and intraguild predation [30,31]. In addition, predator release ratios influenced by foraging capacity of predator, environmental conditions, and spatial distribution of prey are critical for the successful implementation of biological control programs [42].

Previous laboratory studies have independently shown the potential of *X. flavipes*, *C. malaccensis* and *C. eruditus* to manage *L*. *decolor* populations under different temperature and relative humidity conditions and predator–prey (P-P) ratios [20,26]. However, there is a dearth of information on the direct comparative assessment of suppression efficacy and progeny production capabilities of these three predators. A comparative assessment is essential for predator selection and use based on specific conditions in grain storage environments. Therefore, the objective of the current evaluation was to conduct a comparative assessment of the potential of *X. flavipes*, *C. malaccensis* and *C. eruditus* to manage *L*. *decolor* populations under different temperature and relative humidity conditions and P-P ratios using data from two separate studies [20,26]. Specifically, this evaluation compared prey suppression levels and predator progeny production across multiple P-P ratios under varying temperature and relative humidity regimes from the two previous studies. This comparative evaluation provides critical baseline information necessary for effective predator selection and release strategies, and will facilitate the inclusion of these predators into current IPM programs for managing psocids.

## 2. Materials and Methods

### 2.1. Rearing of Liposcelis decolor

Cultures of *L*. *decolor* used as prey for the two previous studies were maintained in laboratory conditions as described in [12,25]. Only adult females of *L. decolor* (hereafter referred to as adult♀ *L. decolor*) that were selected from laboratory cultures were used in both studies.

### 2.2. Rearing of Xylocoris flavipes, Cheyletus eruditus and Cheyletus malaccensis

Cultures of *X. flavipes* were maintained on *L. decolor* under laboratory conditions as described in [13,19]. Only adult females of *X. flavipes* (hereafter referred to as adult♀ *X. flavipes*) were selected and used for the previous study to assess the potential of *X. flavipes* to manage *L*. *decolor* under different temperature and relative humidity conditions and P-P ratios as described in [20]. *Cheyletus eruditus* and *C. malaccensis* cultures used for the previous study on the biocontrol potential of the two predatory mites were maintained under laboratory conditions as described in [12,25,26]. Only adult female predatory mites (hereafter referred to as adult♀ *C*. *eruditus* and adult♀ *C. malaccensis*) were selected and used for the study [26].

### 2.3. Experimental Arenas

The experimental arena for the *X. flavipes* study was a 5.0 cm-diameter basal Petri dish covered by a 5.5 cm-diameter lid (Style Polystyrene, Falcon^®^, Becton Dickinson and Company, Franklin Lakes, NJ, USA), with a total of 54.98 cm^2^ and 29.99 cm^2^ of migration area for the predators and prey, respectively, as described in [13,19,20]. *Liposcelis decolor* were provisioned with 5.0 g of cracked wheat in each basal Petri dish. For the *C. eruditus* and *C. malaccensis* study, the experimental arenas consisted of two 6.0 cm-diameter Petri dishes, forming a total migration area of 113.04 cm^2^ for the predators, whereas prey were confined to a total migration area of 47.12 cm^2^ as described in [12,25,26]. Each basal Petri dish contained 5.0 g of cracked wheat as food for *L. decolor*.

### 2.4. Predation and Progeny Production of Xylocoris flavipes, Cheyletus eruditus and Cheyletus malaccensis

Levels of *L. decolor* population suppression by *X*. *flavipes* were assessed at different predator–prey (P-P) ratios, temperatures and RHs over a 40-day period, as described in [20]. The initial prey density was 240 adult♀ *L. decolor* with five P-P ratios (0:240, 1:240, 2:240, 3:240 or 5:240), four levels of temperature (20, 24, 28 and 32 °C), and three levels of RH (63, 75 and 85%) used. The number of surviving nymphs and adults of *L*. *decolor* were counted to estimate prey suppression by *X*. *flavipes* after 40 days, as described in [20]. In the case of predator progeny production after 40 days, adults and nymphs of *X*. *flavipes* under the four P-P ratios (1:240, 2:240, 3:240 or 5:240) across all temperature and RH combinations were counted as described in [20]. To estimate both prey suppression and progeny production by *X. flavipes*, the total numbers of mobile stages of prey and predator, respectively, in experimental arenas were counted after 40 days. In the *C. eruditus* and *C. malaccensis* study, initial prey density was 20 adult♀ *L. decolor* and suppression levels of the prey were assessed at five P-P ratios (0:20, 1:20, 2:20, 4:20 or 10:20), four levels of temperature (20, 24, 28 and 32 °C), and three levels of RH (63, 75 and 85%) over a 40-day period as described in [26]. Surviving nymphs and adults of *L*. *decolor* were counted to estimate prey suppression by the two predatory mites after 40 days as described in [26]. Again, mobile stages (nymphs and adults) of *C. eruditus* and *C. malaccensis* under four P-P ratios (1:20, 2:20, 4:20 or 10:20) and all temperature and RH combinations were counted and assessed after 40 days as described in [26]. In [26], estimates of percentage prey suppression by *C. malaccensis* and *C. eruditus* is based on total number of mobile stages of prey counted in the experimental arenas after 40 days. Also, in [26], data for *C. malaccensis* and *C. eruditus* progeny production are presented as per capita values of count numbers. However, in this evaluation, to estimate both prey suppression by *C. eruditus* and *C. malaccensis* and progeny production by these two predatory mites, total numbers of mobile stages of prey and predator, respectively, in experimental arenas were counted and used. For both previous studies involving the three predator species, the experimental design was a split-split plot in a randomized complete block design with a 5 x 4 x 3 factorial treatment structure. Factors were P-P ratios with five levels, four levels of temperature and three levels of RH.

### 2.5. Statistical Analysis

To compare the number of *L. decolor* surviving after 40 days of exposure to predators and the number of progeny produced by predators across different P-P ratios, temperatures and RHs, generalized linear mixed model methods were used. For *X*. *flavipes*, the five P-P ratios 0:240, 1:240, 2:240, 3:240 and 5:240 were evaluated, whereas for *C. eruditus* and *C. malaccensis*, the five P-P ratios 0:20, 1:20, 2:20, 4:20 and 10:20 were assessed. All three predators were tested at four temperatures (20, 24, 28 and 32 °C), and three RHs (63, 75 and 85%). Data for *C. eruditus* and *C. malaccensis* from the previous study [26] were reanalyzed from percentage values (percentage prey survival and percentage predator progeny) to actual counts to maintain consistency with analysis used for the *X. flavipes* study [20]. PROC GLIMMIX in SAS models the main effects of P-P ratio, temperature and RH and their interactions for each of the response variables (number of *L. decolor* surviving and number of predator progeny produced) with the specified response distribution (Poisson). For analyses involving percentage reduction in *L. decolor* population relative to control (P-P) ratio, the beta distribution was specified in PROC GLIMMIX. Least-squares means for appropriate significant effects were compared using the Tukey method. All data were analyzed using SAS software version 9.4 (SAS Institute, Cary, NC, USA), and tests were conducted at the nominal 0.05 level of significance.

## 3. Results

### 3.1. Effects of P-P Ratio, Temperature and Relative Humidity on Survival of Liposcelis decolor

Results of tests for the two separate studies showed that the three-way interaction between P-P ratio, temperature and RH significantly affected survival of *L. decolor* when exposed to *X. flavipes* for 40 days (*p* < 0.05), but not when exposed to *C. eruditus* and *C. malaccensis* (*p* > 0.05) (Table 1). However, the two-way interaction between P-P ratio and temperature as well as the main effect of RH were significant for both *C. eruditus* and *C. malaccensis* (*p* < 0.05) (Table 1). In both studies, more prey survived in the control P-P than in ratios containing *X. flavipes*, *C. eruditus* or *C. malaccensis* in all temperature and relative humidity combinations (Table 2, Table 3, Table 4 and Table 5). In the *X. flavipes* study, survival at the control P-P ratio 0:240 was higher than ratios with predators (1:240, 2:240, 3:240 and 5:240) (Table 2). A similar pattern was found in the *C. eruditus* and *C. malaccensis* study, where survival at control P-P ratios 0:20 was consistently higher than P-P ratios of 1:20, 2:20, 4:20 and 10:20 (Table 3 and Table 4). For *C. malaccensis*, the highest P-P ratio, 10:20, was excluded from analysis of the number of surviving *L*. *decolor* because there was near complete prey suppression (more zero prey counts in most temperature–humidity combinations).

**Figure 1:**
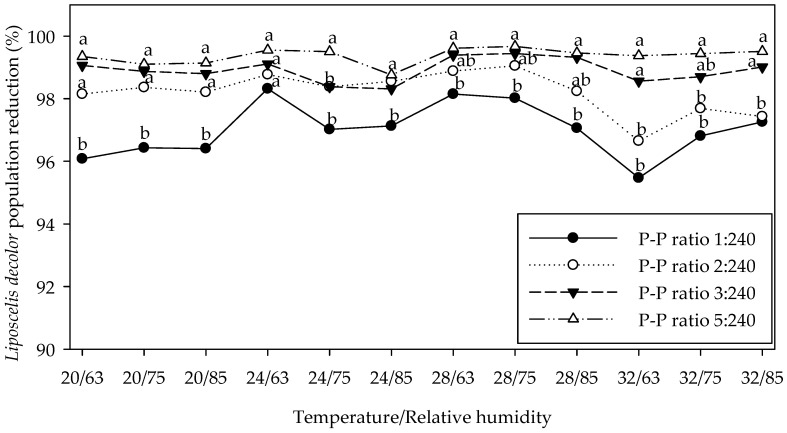
Percentage reduction in *Liposcelis decolor* population relative to control predator–prey (P-P) ratio 0:240 when exposed to *Xylocoris flavipes* at four P-P ratios (1:240, 2:240, 3:240 and 5:240) under four levels of temperature (20, 24, 28 and 32° C) and three levels of relative humidity (RH) (63, 75 and 85%) for 40 days. For each temperature by relative humidity combination, significant differences among P-P ratios are denoted with different letters (*p* < 0.05, SAS, Tukey’s honestly significant difference test).

**Figure 2:**
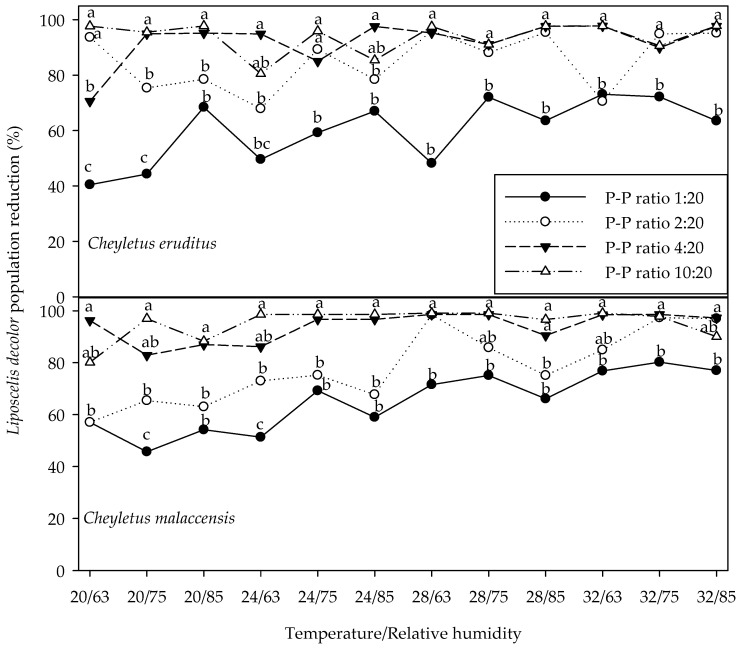
Percentage reduction in *Liposcelis decolor* population relative to control predator–prey (P-P) ratio 0:20 when exposed to *Cheyletus eruditus* and *Cheyletus malaccensis* at four P-P ratios (1:20, 2:20, 4:20 and 10:20) under four levels of temperature (20, 24, 28 and 32° C) and three levels of relative humidity (RH) (63, 75 and 85%) for 40 days. For each temperature by relative humidity combination significant differences among P-P ratios are denoted with different letters (*p* < 0.05, SAS, Tukey’s honestly significant difference test).

Among ratios with predators, prey survival was highest at the lowest P-P ratios across all temperatures and RHs (Table 2, Table 3 and Table 4). Compared with the control P-P ratio, *X. flavipes* significantly suppressed *L. decolor* populations by 97.25%, 98.25%, 99.06% and 99.47% in the 1:240, 2:240, 3:240 and 5:240 P-P ratios, respectively, for the various temperature and RH combinations (Figure 1). Similarly, *C. eruditus* and *C. malaccensis* caused substantial prey reduction. Relative to control P-P 0:20, *C. eruditus* reduced prey populations by 60.15%, 85.29%, 92.28% and 93.76% at 1:20, 2:20, 4:20 and 10:20 P-P ratios, respectively, whereas *C. malaccensis* achieved reductions of 65.28%, 78.35%, 93.88% and 95.23% at the corresponding P-P ratios across the different temperature and humidity combinations (Figure 2). Under temperature and relative humidity conditions of 32 °C and 75% RH, respectively, which represent the optimal environmental conditions for *L. decolor* growth, development and reproduction, prey numbers at the control P-P ratio reached 3985.13 ± 255.45 in the *X*. *flavipes* study, whereas the four P-P ratios with the predator resulted in substantially lower numbers ranging from 19.85 ± 2.47–115.73 ± 8.99. This represents prey reduction of 97.10–99.50% (Table 2; Figure 1). For *C. eruditus* and *C. malaccensis*, prey numbers at the control P-P 0:20 at 32 °C and 75% RH were 46.74 ± 3.43 and 44.15 ± 2.87, respectively (Table 3 and Table 4). However, lower prey numbers of 11.83 ± 2.10, 3.38 ± 1.08, 2.70 ± 0.98 and 2.03 ± 0.84 were found for *C. eruditus* at P-P ratios 1:20, 2:20, 4:20 and 10:20, respectively, and represents 74.69–95.66% reduction (Table 3; Figure 2). For *C*. *malaccensis*, numbers of prey surviving were 7.50 ± 1.58, 2.61 ± 0.93 and 0.98 ± 0.57 at P-P ratios 1:20, 2:20 and 4:20, respectively. This means prey numbers were reduced by 83.01–97.78% compared with control P-P 0:20 (Table 4; Figure 2). The highest P-P ratio 10:20 for *C. malaccensis* almost eliminated all prey and was excluded from statistical analysis of prey survival. Comparatively, despite *X. flavipes* starting with 12-fold more prey than *C. eruditus* and *C. malaccensis*, this predator achieved the highest suppression rates, indicating better predation efficacy.

### 3.2. Effect of P-P Ratio, Temperature and Relative Humidity on Progeny Production by Xylocoris flavipes, Cheyletus eruditus and Cheyletus malaccensis

The three-way interaction of P-P ratio, temperature and RH with regard to predator progeny production after 40 days was not significant for *X. flavipes* (*p* > 0.05) (Table 1). However, the two-way interaction between P-P ratio and temperature was significant (*p* < 0.05), while RH showed no significant effect on progeny production of *X. flavipes* (Table 1). For the *C. eruditus* and *C. malaccensis* study, due to high predator mortality at 63% RH, this RH was excluded from predator progeny analyses. Therefore, only data from 75% and 85% RH across the four temperatures and four P-P ratios (1:20, 2:20, 4:20 and 10:20) were analyzed. Under these conditions, the three-way interaction was significant for *C. eruditus* (*p* < 0.05) but not for *C. malaccensis* (*p* > 0.05) (Table 1). However, for *C. malaccensis*, both the two-way interaction of P-P ratio and RH, and P-P ratio and temperature were significant (*p* < 0.05). In the case of *X. flavipes*, the predator produced more progeny at the P-P ratios of 5:240 and 3:240, in particular at lower temperatures of 20 and 24 °C (Table 6). In contrast, at higher temperatures of 28 and 32 °C, which represent favorable conditions for the prey, progeny production was higher at 1:240 P-P ratio (Table 6). The optimum progeny production by *X*. *flavipes* was observed at a P-P ratio of 1:240 at 28 °C (13.50 ± 1.83) over 40 days.

The progeny production pattern for *C. malaccensis* and *C. eruditus* was different than that of *X. flavipes*. *Cheyletus eruditus* produced a greater number of progeny at 24 °C, 75% RH and P-P ratio 4:20 (42.50 ± 4.56). For *C. malaccensis*, the highest number of progeny was produced at 24 °C and 85% RH with a P-P ratio 4:20 (23.09 ± 7.11). Generally, progeny numbers for both *C. malaccensis* and *C. eruditus* decreased considerably at higher temperatures (28 and 32 °C) compared with lower temperatures (20 and 24 °C) (Table 7).

## 4. Discussion

Psocids are difficult to control in storage facilities worldwide because they have high tolerance and rapidly develop resistance to phosphine and have high reproductive capacity [40,41]. Several natural enemies are reported as potential bioagents for managing psocids [12,13,19,20,25,26]. The current evaluation used data from two separate previous studies to assess and compare the predatory potential of the warehouse pirate bug, *X. flavipes*, and two cheyletid mites, *C. malaccensis* and *C. eruditus* to manage *L. decolor* under different temperature and relative humidity conditions and predator–prey release ratios [20,26].

The comparative evaluation showed that *X*. *flavipes*, *C*. *malaccensis* and *C*. *eruditus* have the potential to manage psocids. However, *X*. *flavipes* can cause greater suppression levels than *C*. *malaccensis* and *C*. *eruditus*. Relative humidity of 63% was detrimental to both *C. malaccensis* and *C. eruditus*. On the contrary, performance of *X. flavipes* was not affected across all RH levels tested, indicating tolerance to diverse RH conditions, in particular those similar to wet subtropical and tropical climates. The different tolerance levels indicate that storage environment conditions can affect performance of biocontrol agents and should be carefully considered in the selection and use of natural enemies in IPM programs. Physical conditions in storage environments, as well as grain moisture content, are known to influence interactions between predators and their prey and can impact the ability of biocontrol agents to suppress pest populations [30,31,43]. Many storage facilities operate at RH levels below 75% RH, particularly in arid regions or in facilities with active ventilation systems [21]. Under conditions of high temperature and low RH, *C. eruditus* and *C. malaccensis* may fail to establish [26]. This makes *X. flavipes*’ tolerance to diverse RH conditions more preferable for practical use in stored-product IPM. Nonetheless, *C. eruditus* is adapted to both temperate and tropical conditions, whereas *C. malaccensis* is more adapted to temperate climates [7,29,44].

Results from the two studies showed that, under favorable conditions, all three predators can exert predation pressure on *L*. *decolor*, with highest prey suppression achieved by *X. flavipes* (>97%) compared with *C. eruditus* and *C. malaccensis* (60.15–95.23% prey suppression). Previous studies have reported that *X. flavipes*, *C. eruditus* and *C. malaccensis* can prey on insect pests of stored grain, including *L*. *decolor*, and can significantly reduce its population [12,13,45,46]. The greater prey suppression levels achieved across all the predator release ratios show predators have high per capita predation rates, causing significant population reductions irrespective of the P-P ratios [12,13,47]. However, suppression of prey population was greater at higher P-P ratios than lower release ratios. The observed predator density-dependent prey suppression has been reported in previous studies [6,20,26]. Between the two cheyletid mites, suppression by *C. malaccensis* was marginally higher in most of the release ratios. This may be explained by the texture of the medium used in the experimental arenas (coarse-wheat grain) which is mostly preferred by *C. malaccensis* [26]. *Cheyletus eruditus* is mostly found in grain residues, whereas *C. malaccensis* is more associated with grain mass because of its capability to penetrate bulk grains [48]. Danso et al. [26] reported that *C. malaccensis* and *C. eruditus* can complete their development on a small number of *L. decolor* and can augment their progeny through parthenogenesis under a wide range of predator release ratios. In addition, the two cheylitid mites are known to adopt cannibalism to survive in the absence of prey. Similarly, *X. flavipes* is known to cannibalize conspecifics when prey is limited [11,20]. Based on the data from the cheyletid mites study, it can be inferred that suppression of *L. decolor* population by *C. malaccensis* and *C. eruditus* would be low compared with *X*. *flavipes* if lower numbers of the predators are released for biological control. Nonetheless, due to density-dependent factors including mutual interference, competition and cannibalism, higher release ratios may result in under-performance of predators [19,20]. Therefore, accurate estimation of the most effective release ratio would be important consideration for successful biological control program [26].

The predatory characteristics of *X*. *flavipes*, *C. eruditus* and *C malaccensis* suggest that they can be released inundatively to provide rapid psocid suppression under certain scenarios in storage environments including bagged commodities, empty storage structures, stacked pallets, and grain residues. In the United States, *X. flavipes* is registered for use against stored-product insect pests, and it can be combined with other natural enemies [21,49,50]. Marketed as Cheyletin^®^, *C. eruditus* is the only commercialized predatory mite approved for use to control mite pests in food storage systems including stored-grain mass, grain residues, debris in seed stores, and empty stores [51]. Commercialization and use of bioagents to suppress insect pests and mite populations in commodity storage environments, including bulk stored-grain, food processing companies, bakeries, seed stores, and empty stores, is common in Europe [11,29,51]. A study in Germany showed that when *Trichogramma evanescens* Westwood (Hymenoptera: Trichogrammatidae) was released at a rate of 25,000 per week and *Habrobracon hebetor* (Say) (Hymenoptera: Braconidae) at a rate of 100 per month, the number of *Ephestia kuehniella* Zeller (Lepidoptera: Pyralidae) found in pheromone traps in a bakery decreased considerably over time [11].

*Xycoris flavipes* and cheyletid mites showed different reproductive responses under their respective test conditions. At higher temperatures, *X*. *flavipes* produced more progeny at the lowest P-P release ratio, whereas at lower temperatures, the predator produced more progeny at the highest P-P release ratio [20]. However, *C. malaccensis* and *C*. *eruditus* increased their progeny production at lower P-P release ratios, lower temperatures, and at higher RH. Unlike in the case of *X*. *flavipes*, survival and population growth of *C. malaccensis* and *C*. *eruditus* was negatively affected by 63% RH [26]. These peculiar trends indicate that *X*. *flavipes* and cheylitid mites use different strategies to optimize reproduction. Because *X. flavipes* is a voracious feeder and also cannibalistic, high metabolic activity at higher temperatures may have resulted in rapid depletion of prey resources to cause conspecific cannibalism at the high release ratios [17,20]. However, at higher temperatures, the lowest release ratio likely maintained sufficient prey to sustain predator progeny production due to less cannibalism [20]. Inoculative releases of *X. flavipes* at low to moderate ratios are recommended for psocid pest management, particularly in warmer storage environments (>28 °C), where such release rates can reduce intraspecific predation and facilitate successful predator population establishment. However, higher release ratios should be used at temperatures below 24 °C to compensate for lower survival of predators [20]. For *C*. *eruditus* and *C. malaccensis*, deploying them in storage facilities with less than their optimal humidity may present a practical challenge because of low survival and reproduction at 63% RH [26]. For example, storage facilities using dehumidification can successfully incorporate *X. flavipes* into psocids management compared with *C. malaccensis* and *C*. *eruditus*.

Overall, because this evaluation is based on data from two independent studies with different initial prey densities, predator–prey ratios, and arena configurations, comparisons between the predators should be interpreted cautiously as methodological differences could have influenced responses.

## 5. Conclusions

This comparative evaluation demonstrated that *X*. *flavipes*, *C*. *eruditus* and *C. malaccensis* have potential to manage *L*. *decolor* populations. *X*. *flavipes* achieved prey suppression above 97% across all predator–prey release ratios and environmental conditions tested, with maximum suppression of 99.47% at the highest release ratio (5:240), whereas *Cheyletus eruditus* and *C. malaccensis* reduced prey population by 60.15–93.76% and 65.28–95.23%, respectively, across the tested P-P ratios. Temperature significantly influenced progeny production for all three predators with optimal progeny production occurring at higher temperatures (28–32 °C) for *X*. *flavipes* and at lower temperatures (20–24 °C) for *C. eruditus* and *C. malaccensis*. Relative humidity of 63% decreased survival and reproduction of *C. eruditus* and *C. malaccensis* whereas *X. flavipes* performance was not affected across the RH levels. To minimize conspecific predation and ensure good predator establishment in biocontrol applications, lower to medium P-P release ratios should be considered. Field validation involving wider range of release ratios and evaluation of predator sustainability is required. In addition, evaluation of the three predators against other economically important *Liposcelis* species including *L*. *entomophila*, *L*. *bostrychophila* and *L*. *paeta*, compatibility of *X*. *flavipes* with *C*. *eruditus* and *C. malaccensis*, and the impact of pesticides on predators should be investigated to enable integration of these three predators into storage IPM systems for management of psocids.

## Figures and Tables

**Table 1:** Summary of tests for main effects of predator–prey (P-P) ratio, temperature (T) and relative humidity (RH), and interactions for number of prey surviving (*Liposcelis decolor*) and predator progeny production of *Xylocoris flavipes*, *Cheyletus eruditus* and *Cheyletus malaccensis* over 40 days. Initial prey density for *X*. *flavipes* was 240 females of *L*. *decolor*, and that for *C. eruditus* and *C. malaccensis* was 20 females of *L*. *decolor*.

Variable	Predator spp.	Source	DF	*F*	*p* Value
Prey survival	*X*. *flavipes*	T	3, 15	57.42	<0.0001
		RH	2, 40	71.92	<0.0001
		T*RH	6, 40	5.77	0.0002
		P-P	4, 144	19904.00	<0.0001
		T*P-P	12, 144	32.00	<0.0001
		RH*P-P	8, 144	5.60	<0.0001
		T*RH*P-P	24, 144	11.14	<0.0001
	*C*. *eruditus*	T	3, 15	1.16	0.3575
		RH	2, 40	11.53	0.0001
		T*RH	6, 40	2.39	0.0455
		P-P	4, 96	174.13	<0.0001
		T*P-P	12, 96	2.54	<0.0059
		RH*P-P	8, 96	0.74	<0.6572
		T*RH*P-P	24, 96	0.85	0.6640
	*C*. *malaccensis*	T	3, 15	1.18	0.3502
		RH	2, 40	13.53	<0.0001
		T*RH	6, 40	0.90	0.5054
		P-P	3, 72	214.39	<0.0001
		T*P-P	9, 72	5.42	<0.0001
		RH*P-P	6, 72	0.67	0.6754
		T*RH*P-P	18, 72	0.54	0.9313
Predator progeny	*X*. *flavipes*	T	3, 9	9.58	0.0037
		RH	2, 24	2.51	0.1025
		T*RH	6, 24	1.82	0.1373
		P-P	3, 108	2.83	0.0419
		T*P-P	9, 108	3.63	0.0005
		RH*P-P	6, 108	0.31	0.9317
		T*RH*P-P	18, 108	0.47	0.9663
	*C*. *eruditus*	T	3, 6	48.16	0.0001
		RH	1, 8	1.38	0.2739
		T*RH	3, 8	10.18	0.0042
		P-P	3, 48	25.11	<0.0001
		T*P-P	9, 48	9.89	<0.0001
		RH*P-P	3, 48	9.63	<0.0001
		T*RH*P-P	19, 48	10.39	<0.0001
	*C*. *malaccensis*	T	3, 6	17.45	0.0023
		RH	1, 8	13.84	0.0059
		T*RH	3, 8	0.52	0.6828
		P-P	3, 33	12.76	<0.0001
		T*P-P	9, 33	6.50	<0.0001
		RH*P-P	3, 33	7.71	0.0005
		T*RH*P-P	7, 33	1.64	0.1586

Asterisk (*) denotes interactions between variables predator–prey (P-P) ratio, temperature (T) and relative humidity (RH).

**Table 2:** Mean number of *Liposcelis decolor* surviving (±SE) over 40 days. Predator was *Xylocoris flavipes*, initial prey density was 240 females of *L. decolor*, five levels of predator–prey (P-P) ratio (0:240, 1:240, 2:240, 3:240 and 5:240), four levels of temperature (T) (20, 24, 28 and 32 °C) and three levels of relative humidity (RH) (63, 75 and 85%).

Temperature (T)	Relative Humidity (RH)	Predator–Prey (P-P) Ratio				
		0:240	1:240	2:240	3:240	5:240
20	63	772.60 ± 50.56 aD	32.84 ± 3.60 bE	15.00 ± 2.19 cF	7.24 ± 1.45 dE	5.69 ± 1.27 dE
20	75	1134.33 ± 73.64 aC	40.43 ± 4.08 bD	18.38 ± 2.43 cE	12.01 ± 1.88 dD	9.31 ± 1.62 dE
20	85	1140.50 ± 74.03 aC	42.35 ± 4.26 bD	20.54 ± 2.64 cE	13.19 ± 2.02 dD	9.89 ± 1.71 dE
24	63	1850.06 ± 119.29 aB	31.48 ± 3.46 bE	22.41 ± 2.78 cE	15.86 ± 2.24 dD	7.30 ± 1.43 eE
24	75	2176.50 ± 140.10 aB	62.59 ± 5.56 bC	35.90 ± 3.66 cC	15.65 ± 2.17 dD	10.43 ± 1.71 dD
24	85	2028.08 ± 130.64 aB	60.93 ± 5.55 bC	29.70 ± 3.35 cD	37.38 ± 3.91 cB	24.83 ± 2.98 dA
28	63	1850.68 ± 119.33 aB	34.00 ± 3.65 bE	21.66 ± 2.72 cE	11.59 ± 1.86 dD	6.54 ± 1.35 eE
28	75	3340.82 ± 214.36 aA	61.60 ± 5.47 bC	29.17 ± 3.21 cD	16.80 ± 2.25 dD	7.93 ± 1.45 eE
28	85	3393.07 ± 217.69 aA	98.79 ± 8.01 bA	59.32 ± 5.38 cB	23.29 ± 2.82 dC	16.67 ± 2.29 eC
32	63	1282.16 ± 83.07 aC	58.49 ± 5.31 bC	44.77 ± 4.37 cC	19.50 ± 2.50 dC	7.70 ± 1.45 eE
32	75	3985.13 ± 255.45 aA	115.73 ± 8.99 bA	82.79 ± 6.83 cA	45.57 ± 4.33 dA	19.85 ± 2.47 eB
32	85	3512.91 ± 225.33 aA	88.54 ± 7.25 bB	82.53 ± 6.85 bA	31.44 ± 3.36 cB	14.10 ± 2.01 dD

Significant differences among P-P ratios for each T*RH combination are denoted with different lower-case letters (within the same row) and differences among T*RH combinations for each P-P ratio are denoted by different upper-case letters (within column), (*p* < 0.05, SAS, Tukey’s honestly significant difference test).

**Table 3:** Mean number of *Liposcelis decolor* surviving (±SE) over 40 days. Predator was *Cheyletus eruditus*, initial prey density was 20 females of *L. decolor*, five levels of predator–prey (P-P) ratio (0:20, 1:20, 2:20, 4:20 and 10:20), four levels of temperature (T) (20, 24, 28 and 32 °C) and three levels of relative humidity (RH) (63, 75 and 85%).

Temperature (T)	Relative Humidity (RH)	Predator–Prey (P-P) Ratio				
		0:20	1:20	2: 20	4:20	10:20
20	63	7.16 ± 1.13 aD	4.32 ±1.22 bB	1.99 ± 0.82 cB	1.99 ± 0.82 cB	0.33 ± 0.33 c
20	75	11.83 ± 1.49 aC	6.56 ± 1.52 bB	3.28 ± 1.05 AbB	1.64 ± 0.74 cB	0.98 ± 0.57 c
20	85	14.82 ± 1.69 aC	4.23 ± 1.20 bB	2.60 ± 0.93 bB	1.63 ± 0.73 cB	0.33 ± 0.33 c
24	63	8.50 ± 1.24 aD	4.30 ± 1.22 bB	1.99 ± 0.82 cB	1.32 ± 0.67 cB	1.32 ± 0.67 c
24	75	12.66 ± 1.55 aC	4.85 ± 1.28 bB	2.59 ± 0.93 bB	1.29 ± 0.65 cB	0.65 ± 0.46 c
24	85	13.15 ± 1.58 aC	3.89 ± 1.45 bB	2.60 ± 0.93 bB	0.97 ± 0.57 cB	1.30 ± 0.65 c
28	63	8.99 ± 1.28 aD	4.60 ± 1.26 bB	2.63 ± 0.94 bB	0.99 ± 0.57 cB	0.99 ± 0.57 c
28	75	41.41 ± 3.17 aA	10.70 ± 2.01 bA	2.42 ± 0.92 cB	1.73 ± 0.78 cB	1.73 ± 0.78 c
28	85	19.16 ± 1.97 aB	6.59 ± 1.52 bB	3.29 ± 1.06 bA	0.99 ± 0.57 cB	0.66 ± 0.47 c
32	63	14.15 ± 1.65 aC	3.42 ± 1.10 bB	0.68 ± 0.49 cB	0.34 ± 0.34 cB	0.34 ± 0.34 c
32	75	46.74 ± 3.43 aA	11.83 ± 2.10 bA	3.38 ± 1.08 cA	2.70 ± 0.98 cA	2.03 ± 0.84 c
32	85	22.16 ± 2.14 aB	7.60 ± 1.64 bA	1.98 ± 0.82 cB	0.99 ± 0.57 cB	0.33 ± 0.33 c

Significant differences among P-P ratios for each T*RH combination are denoted with different lower-case letters (within the same row) and differences among T*RH combinations for each P-P ratio are denoted by different upper-case letters (within column), (*p* < 0.05, SAS, Tukey’s honestly significant difference test).

**Table 4:** Mean number of *Liposcelis decolor* surviving (±SE) over 40 days. Predator was *Cheyletus malaccensis*, initial prey density was 20 females of *L. decolor*, four levels of predator–prey (P-P) ratio (0:20, 1:20, 2:20 and 4:20), four levels of temperature (T) (20, 24, 28 and 32 °C), and three levels of relative humidity (RH) (63, 75 and 85%).

Temperature (T)	Relative Humidity (RH)	Predator–Prey (P-P) Ratio			
		0:20	1:20	2: 20	4:20
20	63	8.83 ± 1.23 aE	3.60 ± 1.09 bB	2.29 ± 0.87 bB	1.31 ± 0.66 b
20	75	12.83 ± 1.49 aD	6.87 ± 1.51 bA	4.25 ± 1.19 bA	1.96 ± 0.80 c
20	85	14.66 ± 1.59 aD	6.54 ± 1.47 bA	5.56 ± 1.36 bA	1.64 ± 0.73 c
24	63	11.66 ± 1.42 aD	5.54 ± 1.35 bA	2.93 ± 0.98 cB	1.30 ± 0.65 c
24	75	18.99 ± 1.82 aD	5.54 ± 1.35 bA	4.57 ± 1.23 bA	1.96 ± 0.80 c
24	85	18.16 ± 1.78 aD	7.18 ± 1.54 bA	5.54 ± 1.35 bA	1.96 ± 0.80 c
28	63	11.99 ± 1.44 aD	3.26 ±1.04 bB	0.98 ± 0.57 cC	0.33 ± 0.33 c
28	75	33.15 ± 2.46 aB	7.50 ± 1.58 bA	3.91 ± 1.14 bA	0.98 ± 0.57 c
28	85	22.99 ± 2.02 aC	7.50 ± 1.58 bA	5.22 ± 1.31 bA	1.63 ± 0.73 c
32	63	15.16 ± 1.62 aD	3.26 ± 1.04 bB	1.96 ± 0.80 bB	0.98 ± 0.57 b
32	75	44.15 ± 2.87 aA	7.50 ± 1.58 bA	2.61 ± 0.93 cB	0.98 ± 0.57 c
32	85	26.99 ± 2.22 aB	5.87 ± 1.39 bA	1.96 ± 0.80 cB	1.30 ± 0.65 c

Significant differences among P-P ratios for each T*RH combination are denoted with different lower-case letters (within the same row) and differences among T*RH combinations for each P-P ratio are denoted by different upper-case letters (within column), (*p* < 0.05, SAS, Tukey’s honestly significant difference test).

**Table 5:** Summary of tests for main effects of predator–prey (P-P) ratio, temperature (T) and relative humidity (RH), and interactions for percentage reduction in *Liposcelis decolor* population relative to control P-P ratio 0:240 when exposed to *Xylocoris flavipes*, or control P-P ratio 0:20 when exposed to *Cheyletus eruditus* and *Cheyletus malaccensis* for 40 days.

Variable	Predator spp.	Source	Df	*F*	*p* Value
Prey reduction (%)	*X*. *flavipes*	T	3, 144	17.60	<0.0001
		RH	2, 144	3.55	<0.0312
		T*RH	6, 144	5.25	<0.0001
		P-P	3, 144	160.42	<0.0001
		T*P-P	9, 144	4.24	<0.0001
		RH*P-P	6, 144	0.75	0.6075
		T*RH*P-P	18, 144	11.14	0.8408
	*C*. *eruditus*	T	3, 96	4.61	0.0047
		RH	2, 96	2.80	0.0656
		T*RH	6, 96	1.52	0.1808
		P-P	3, 96	62.71	<0.0001
		T*P-P	9, 96	2.27	0.0240
		RH*P-P	6, 96	2.42	0.0323
		T*RH*P-P	18, 96	2.22	0.0069
	*C*. *malaccensis*	T	3, 96	34.28	<0.0001
		RH	2, 96	4.95	0.0090
		T*RH	6, 96	2.79	0.0153
		P-P	3, 96	93.82	<0.0001
		T*P-P	9, 96	3.55	0.0008
		RH*P-P	6, 96	0.64	0.6998
		T*RH*P-P	18, 96	2.95	0.0003

Asterisk (*) denotes interactions between variables predator–prey (P-P) ratio, temperature (T) and relative humidity (RH).

**Table 6:** Mean number of progeny (±SE) of *Xylocoris flavipes* over 40 days. Initial prey density was 240 females of *Liposcelis decolor*, four levels of predator–prey (P-P) ratio (1:240, 2:240, 3:240 and 5:240), four levels of temperature (T) (20, 24, 28, and 32 °C) and three levels of relative humidity (RH) (63, 75 and 85%).

Temperature (T)	Relative Humidity (RH)	Predator–Prey (P-P) Ratio			
		1:240	2:240	3:240	5:240
20	63	3.50 ± 0.94 Db	5.75 ± 1.20 aB	5.25 ± 1.15 aB	7.00 ± 1.32 a
20	75	4.25 ± 1.03 bD	5.75 ± 1.20 aB	6.50 ± 1.27 aAB	7.75 ± 1.39 a
20	85	3.75 ± 0.97 bD	3.50 ± 0.93 bC	5.00 ± 1.12 aB	7.00 ± 1.32 a
24	63	6.50 ± 1.27 bBC	7.50 ± 1.37 bAB	8.50 ± 1.46 abA	9.50 ± 1.54 a
24	75	3.50 ± 0.93 bD	5.00 ± 1.12 aB	6.25 ± 1.25 aAB	7.75 ± 1.39 a
24	85	5.25 ± 1.15 aC	4.75 ± 1.09 aB	5.50 ± 1.17 aB	6.50 ± 1.27 a
28	63	13.50 ± 1.83 aA	7.75 ± 1.39 bAB	7.25 ± 1.35 bAB	8.00 ± 1.41 b
28	75	10.00 ± 1.58 aB	8.00 ± 1.41 aAB	6.00 ± 1.22 aAB	7.25 ± 1.35 a
28	85	9.00 ± 1.50 aB	6.50 ± 1.27 abAB	5.75 ± 1.20 bB	7.50 ± 1.37 ab
32	63	8.75 ± 1.48 aB	6.00 ± 1.22 bAB	5.50 ± 1.17 bB	8.00 ± 1.41 a
32	75	9.00 ± 1.50 aB	9.00 ± 1.50 aA	5.75 ± 1.20 bB	7.25 ± 1.35 ab
32	85	7.75 ± 1.39 aBC	7.50 ± 1.37 aAB	7.75 ± 1.39 aAB	7.00 ± 1.32 a

Significant differences among P-P ratios for each T*RH combination are denoted with different lower-case letters (within the same row) and differences among T*RH combinations for each P-P ratio are denoted by different upper-case letters (within column) (*p* < 0.05, SAS, Tukey’s honestly significant difference test).

**Table 7:** Mean number of progeny (±SE) of *Cheyletus eruditus* and *Cheyletus malaccensis* over 40 days. Initial prey density was 20 females of *Liposcelis decolor*, four levels of predator–prey (P-P) ratio (1:20, 2:20, 4:20 and 10:20), four levels of temperature (T) (20, 24, 28 and 32 °C) and two levels of relative humidity (RH) (75 and 85%).

Predator	Temperature (T)	Relative Humidity (RH)	Predator–Prey (P-P) Ratio			
			1:20	2: 20	4:20	10:20
*C. eruditus*	20	75	13.65 ± 2.29 bB	16.32 ± 2.53 bB	13.32 ± 2.26 bC	32.63 ± 3.84 aA
	20	85	11.99 ± 2.13 B	15.32 ± 2.44 B	12.32 ± 2.16 C	13.32 ± 2.26 B
	24	75	12.29 ± 2.15 cB	28.22 ± 3.51 bA	42.50 ± 4.56 aA	18.93 ± 2.76 cB
	24	85	27.99 ± 3.49 A	34.67 ± 3.99 A	31.00 ± 3.72 B	31.67 ± 3.77 A
	28	75	11.00 ± 2.03 aB	8.66 ± 1.78 bC	8.33 ± 1.74 bC	14.66 ± 2.38 aB
	28	85	3.67 ± 1.13 bC	7.33 ± 1.63 aC	10.00 ± 1.92 aC	11.67 ± 2.09 aB
	32	75	4.98 ± 1.32 bC	2.32 ± 0.89 bD	4.65 ± 1.27 bD	38.51 ± 4.27 aA
	32	85	12.64± 2.19 B	10.31 ± 1.95 C	14.97 ± 2.41 C	14.30 ± 2.35 B
*C. malaccencis*	20	75	5.31 ± 1.63 bB	5.61 ± 1.73 bB	9.53 ± 2.93 bB	18.24 ± 5.61 aA
	20	85	7.16 ± 2.20 bB	12.74 ± 3.92 aA	13.47 ± 4.16 aB	14.95 ± 4.60 aA
	24	75	3.71 ± 1.14 C	9.68 ± 2.98 B	17.30 ± 5.32 A	19.43 ± 5.98 A
	24	85	15.06 ± 4.64 bA	15.69 ± 4.83 bA	23.09 ± 7.11 aA	19.81 ± 6.10 aA
	28	75	0.77 ± 0.37 cD	1.85 ± 0.89 bD	2.55 ± 0.92 bD	11.76 ± 3.62 aB
	28	85	4.50 ± 1.39 aB	3.14 ± 0.97 bC	6.42 ± 1.98 aC	6.98 ± 2.15 aB
	32	75	3.51 ± 1.27 C	-	2.02 ± 0.97 D	2.05 ± 0.63 C
	32	85	6.37 ± 1.96 aB	1.08 ± 0.52 bC	-	1.85 ± 0.67 bC

Significant differences among P-P ratios for each T*RH combination are denoted with different lower-case letters (within the same row) and differences among T*RH combinations for each P-P ratio are denoted by different upper-case letters (within column), (*p* < 0.05, SAS, Tukey’s honestly significant difference test).

## Data Availability

The original contributions presented in this study are included in the article. Further inquiries can be directed to the corresponding author.
